# Tuberculosis Treatment Adherence of Patients in Kosovo

**DOI:** 10.1155/2017/4850324

**Published:** 2017-11-05

**Authors:** Shaip Krasniqi, Arianit Jakupi, Armond Daci, Bahri Tigani, Nora Jupolli-Krasniqi, Mimoza Pira, Valbona Zhjeqi, Burim Neziri

**Affiliations:** ^1^Institute of Pharmacology and Toxicology and Clinical Pharmacology, Faculty of Medicine, University of Prishtina “Hasan Prishtina”, Prishtina, Kosovo; ^2^Health Agency for Development (HAD), Prishtina, Kosovo; ^3^Department of Pharmacy, Faculty of Medicine, University of Prishtina “Hasan Prishtina”, Prishtina, Kosovo; ^4^National Institute of Public Health of Kosovo, Prishtina, Kosovo; ^5^Institute of Clinical Physiology, Faculty of Medicine, University of Prishtina “Hasan Prishtina”, Prishtina, Kosovo

## Abstract

**Setting:**

The poor patient adherence in tuberculosis (TB) treatment is considered to be one of the most serious challenges which reflect the decrease of treatment success and emerging of the Multidrug Resistance-TB (MDR-TB). To our knowledge, the data about patients' adherence to anti-TB treatment in our country are missing.

**Objective:**

This study was aimed to investigate the anti-TB treatment adherence rate and to identify factors related to eventual nonadherence among Kosovo TB patients.

**Design:**

This study was conducted during 12 months, and the survey was a descriptive study using the standardized questionnaires with total 324 patients.

**Results:**

The overall nonadherence for TB patient cohort was 14.5%, 95% CI (0.109–0.188). Age and place of residence are shown to have an effect on treatment adherence. Moreover, the knowledge of the treatment prognosis, daily dosage, side effects, and length of treatment also play a role. This was also reflected in knowledge regarding compliance with regular administration of TB drugs, satisfaction with the treatment, interruption of TB therapy, and the professional monitoring in the administration of TB drugs.

**Conclusion:**

The level of nonadherence TB treatment in Kosovar patients is not satisfying, and more health care worker's commitments need to be addressed for improvement.

## 1. Introduction

Tuberculosis (TB) remains still one of the most important socioeconomic global diseases with high infection and mortality rate [1]. The World Health Organization (WHO), in 2015, reported worldwide 10.4 million people infected with TB and annually 1.5 million patients' death [2, 3].

According to WHO reports, from a total number of infected people with TB, 123 000 cases were diagnosed and reported as multidrug-resistant TB (MDR-TB). Nevertheless, the occurrence of MDR-TB is estimated to be almost four times higher, showing that detection and report of such cases are not in the expected level [2, 4].

The TB is considered as a global human threat and is included in United Nations (UN) millennium goals to reduce the risk from this disease [5]. According to these goals, there are still needs for more improvement, especially in the international coordinated plan to reduce the mortality rate, the incidence of MDR-RB patients, and risk from this disease overall [6].

Despite many achievements in the fight against TB, this disease is still challenging the human health [7].

The global initiatives to fight TB are determined by WHO strategy, directly observed therapy short course (DOTS), and one of the most important components of this strategy is the effective TB drug supply and management system. One of the important issues that guarantee the success of the treatment is the degree of anti-TB drug patient's compliance or adherence. The poor patient adherence is considered to be one of the most serious challenges which reflects the decrease of treatment success and emerging of the MDR-TB [8–10]. Directly observed therapy (DOT) was outlined to support TB treatment adherence and completion, of course, resulting in reduced morbidity and mortality and restraining TB drug resistance.

Although the implementation of DOTS strategy improved the treatment success of TB patients, there is evidence showing that direct observation is not implemented systematically and level of patient's adherence to TB treatment is not satisfying (from 4 = 50% in India, 88.5% in Ethiopia, and 88.8% in China) [11–13].

Kosovo, the state in Southeastern Europe, has a relatively high number of TB cases in this region [14]. Until 2000, the ratio of the rural versus urban general population in Kosovo was 62% versus 38% [15], while after this period massive migration happened from rural regions. The Global Fund to Fight AIDS, Tuberculosis, and Malaria supported Kosovo with grants which resulted in substantial reduction in a number of TB cases (from 85.9/100,000 in 2000 to 46/100,000 inhabitants in 2012) [16].

Even with this improvement, Kosovo remains among states with highest notification rates of TB in the region and more attention is needed. To our knowledge, the data about patients' adherence to anti-TB treatment in our country are missing; therefore, this study was aimed to investigate the anti-TB treatment adherence rate and to identify factors related to eventual nonadherence among Kosovo TB patients.

## 2. Material and Methods

### 2.1. Setting and Design

It was a descriptive study conducted for 12 months using standardized questionnaires. The interview was conducted for 324 TB patients.

### 2.2. Sample Size

According to data of the National Tuberculosis Control Program in Kosovo, in 2012, 968 new and recurrent/relapsed TB patients were registered. From this total number of active TB patients in this year, we have interviewed 350 patients in the survey. A total number of 324 were completely responsive, while 26 patients answered partially which we have excluded from data analysis. TB patients were recruited by using a systematic sampling method after fulfillment of inclusion criteria such as a registered TB patients with confirmed diagnosis of TB and being regularly supplied with TB drugs.

The nonadherence was classified as follows: patients who did not take the TB drugs for more than three days were considered nonadherent to TB drugs.

Data were obtained from TB out-patient registers from TB dispensaries in six administrative regions of Kosovo. The TB health centers are mainly located in the distance less than 10 km for each region, ensuring easy access for patients to these TB centers.

The random methodology was used to recruit study participants to assess the level of adherence to anti-TB treatment. We have recruited each third patient found in TB out-patient register. A sample size of 324 reached the target to estimate the level of TB drug adherence for 90% level of adherence with a 5% margin of error and 95% confidence.

The main indicator to allocate the TB patient adherent or nonadherent was the fact if the patient has forgotten to receive the TB drugs in a period longer than three days.

The patients who were not mentally capable of answering due to accompanied mentally diseases during the interview were excluded from the study. From 350 interviewed patients, 26 patients were excluded due to incorrect answers.

The cohort of TB patients was monthly supervised by the pulmonary doctor. Indeed, in a monthly basis, the TB patients visit the TB dispensary for TB drug supply and undergo the medical evaluation by the pulmonary doctor.

### 2.3. Management of Data

The collection of the data has been performed using the standardized questionnaires, which initially were standardized to yield the reliable results of TB treatment adherence.

In this study, we have enrolled the responders from all medical centers that provide health care for TB patients in Kosovo's health system which has ensured the distributed representation of TB patients from all regions. Initially, during the visits, the research team stayed at these centers during working hours, and they accessed the documentation of all the patients that received anti-TB therapy and start to trace these patients to perform the individual interviews. Patients' knowledge about TB treatment adherence was ascertained based on their responses obtained during a face-to-face interview.

The questionnaire was composed of five chapters: (a) sociodemographic; (b) level of knowledge on tuberculosis disease; (c) knowledge on tuberculosis treatment; (d) TB treatment adherence characteristics, and (e) health system and other features.

### 2.4. Statistical Analysis

We used a descriptive and comparative statistics. The differences between groups and the proportion of poor adherence for all variables were analyzed using chi-square *χ*2 test and minimum significant level of *p* < 0.05. All statistical analysis was performed using the Graph Prism 6.0 (Software).

### 2.5. Ethical Issue

The study protocol was approved by Country Coordinating Mechanisms (CCM) established by the Ministry of Health of Kosovo. Initially, we present the aim of this survey and other relevant information to TB patients. The verbal consent was obtained, taking into consideration the fact that we have expected a relative number of patients with low-level literacy, with elementary school education (37.04%), and who are not literate (14.81%), which might influence their ability to understand and to sign the document. Data collected by interview were managed anonymously using the codes and no names.

## 3. Results

A total of 350 TB patients were interviewed, while only 324 completed the interview (response rate of 92.6%). About half of TB patients (49.38%) enrolled in the study were male while other patients were female (50.4%). Based on age, the patients included in survey have been divided into seven groups as follows: in age group 0–15 years (4.01%), followed by a group of 16–25 (29.01%), 26–35 (12.35%), 36–45 (11.73), 46–55 (11.73%), 56–65 (12.04), and >65 years old (19.14).

Most TB patients were not married (59.88%) and had elementary (37.04%) and high school education (38.27%) with higher unemployed rates (64.51%) (Table 1).

Moreover, the knowledge of patients about TB disease showing the level of health education about TB was shown in Table 2. According to DOTS, all these patients had undergone the health education module for TB disease. Mostly respondents do not know or had a wrong answer regarding what causes TB (56.18% and 22.22%, resp.). We get the similar structure of answers in questions how TB is spread and how TB is prevented where the cohort of patients with answers do not know or not true were higher compared to patients with the exact answer (62.65% and 72.22 versus 37.35% and 27.78%, resp.), while the majority of TB patients had a correct answer compared to those with an incorrect answer to question is TB cured (88.27% versus 11.73%).

However, the knowledge about TB therapy is represented in Table 3. Following this issue, the highest structure of respondents answered correctly for the length of TB treatment and about colors of TB drugs (75.31% and 65.43%). In majority cases, they answered not correctly about names of TB drugs, the daily dosage of TB drugs, and knowledge about the side effect of TB drugs (86.73%, 72.22%, and 76.85%).

The comprehensive data about knowledge for TB drug compliance and risk behavior of TB patients were shown in Table 4. These results highlighted different variables reflecting the optimal knowledge about TB treatment compliance of TB patients registered in our study.

Almost 81.17% of patient answered correctly for the importance of regular administration of TB drugs, 73.15% reported a regular supply of free TB drugs, 95.3% of those were satisfied with treatment in the health center, and 68.52% of patients have a health center in a distance less than 10 km. We had only 1.85% of patients drinking alcohol, while 11.42% were smokers. The unfavorable finding was the structure of patients with interruption of TB therapy after three months, due to a nonregular intake of drugs by them because of not respecting the pulmonary doctor's advice (14.5%).

The overall adherence and nonadherence for TB patient cohort included in the survey were 85.5% (95% CI 0.812–0.892) and 14.5% (95% CI 0.109–0.188), respectively.

The major factors such as the knowledge about the daily dosage of TB drug were shown to be a difference between adherent and nonadherent group (*p* < 0.0001). Also, the knowledge about the length of TB treatment and side effect of TB drugs is significantly different between adherent and nonadherent group (*p* < 0.018 and *p* < 0.007, resp.). Our results show that the other variables in this context had no effects on TB treatment adherence (Table 3).

The most important variables with the highest level of significance in TB treatment adherence were the regular administration of TB drugs (*p* < 0.001), satisfaction with the treatment in the health center (*p* < 0.001), and interruption of TB therapy after three months (*p* < 0.001).

Also, the presence of a nurse or family member in the administration of TB drugs was considered to be different between the compared groups (*p* < 0.039) (Table 4). Other variables presented in Table 4 had no substantial significance in TB treatment adherence in patients involved in our study.

However, minor influencing factors for nonadherence to anti-TB treatment such as variables of knowledge about TB disease did not show a significant effect on the TB treatment adherence, while the impact of knowledge about the question regarding the TB treatment had a significant impact on TB treatment adherence (*p* < 0.001) (Table 2). Moreover, there was a significant difference between the adherent and nonadherent group's age (*p* < 0.018) with an increased nonadherence level in ages from 36 to 65 years old compared to adherence levels as well as place of residence with an increased nonadherence level in urban compared to the rural place of residence (*p* < 0.015), while other variables such as sex, marital status, education, and employment did not affect TB treatment adherence (Table 1).

## 4. Discussion

Besides intensive implementation of TB-DOTS control strategy, the treatment adherence for tuberculosis infection remains suboptimal in the majority of countries with a moderate and high prevalence of TB disease [3].

The treatment compliance with antituberculosis drugs is an essential part of DOTS and is mandatory for achieving the TB treatments' success [17].

Nonadherence to anti-TB treatment might trigger TB drug resistance which prolongs the infectiousness of disease and increases the relapse and death of patients [18].

The official registered number of TB patients in Kosovo during 2012 was 968 [14]. Our study results indicate that, from 324 interviewed TB patients, 85.5% adhered to the TB treatment regimen. This TB treatment adherence is lower than what was reported in Tanzania (95%) [19] and similar with results reported in Uganda (92%) [20]. The treatment adherence in our study was better to compare to adherence reported from several studies conducted in different countries with high prevalence of TB disease, such as in Iran (70%) [21], Ethiopia (79%) [22], and India (59.5%) [23].

Data from the literature present that the incidence of tuberculosis is usually higher in males than female TB patients. In our study, the genders are almost equally represented among cohort of patients with no impact on TB treatment adherence. Similar results about gender distribution found in TB patients are also reported in other related studies [24, 25].

The ratio of TB population in urban and rural areas is approximately 1.1 to 0.9 and has a significant effect on TB patient adherence, while in a research report published in Holland the ratio of the TB patients in urban and rural areas was 3.8 to 1 [26]. A dominance of TB patients in urban areas is also reported in other countries in Europe [27]. Taking into consideration that, in our country, in the last two decades there was a massive migration of population from rural towards urban locations, this phenomenon reflected in the ratio of TB patients between urban and rural regions (61.42% versus 38.58%) was registered in our survey.

Furthermore, urban location was shown to have a similar number of adherents comparing to total urban versus rural patients and a higher number of nonadherent patients compared to rural location.

The majority of the patients were from 16 to 25 years old and not married. This group of patients usually is young people living a sedentary life alone without family care. The minor influencing factors including age group show a significant difference in TB treatment adherence while marital status had no impact.

We also found that education level and employment do not play a role in TB treatment adherence. Nevertheless, the educational attainment usually is correlated with patient's TB knowledge and seems to have an impact on TB treatment adherence, while the employment does not have an effect [22, 28].

There are also reports that educational level, type or residence, manufacturing industries, and service sectors were associated with poor adherence in the developed countries [29]. In general, we have shown that from the sociodemographic variables in our study only age group and location influenced the level of TB treatment adherence.

We found that a high number of TB patients answered not properly about causes of TB disease, its contagious rate, and TB prevention, reflecting the lack of basic knowledge about the disease. All these variables were without impact between adherent and nonadherent patients, while the knowledge about whether TB is cured was significant between these two groups of patients. Similar results found other researchers presenting inadequate level of TB knowledge in patients in Equatorial Guinea [28] and Ethiopia [30].

Moreover, our results regarding major influencing factors including knowledge about TB treatments including daily dosages, the length of treatment, and side effects have shown to play a role in treatment adherence. The level of knowledge for TB treatment in our study is not sufficient and can be due to non-well-established communication between health staff and patients and due to incorrect perception and misunderstanding of information received from health care professionals of TB patients about treatment. These findings are in line also with other studies which also recommend more attention to improve the communication with TB patients [31, 32].

The analyses of knowledge for TB compliance yielded the lack of knowledge between adherent and nonadherent patients in several important and variables such as importance of regular administration of TB drugs, satisfaction with treatment in the health centers, interruption of therapy after three months, and administration of TB therapy in the monitoring of nurse or family members. These variables represent a core of direct indicators and major influencing factors for TB adherence and its consensus that they are the most useful set of indicators for evaluation of TB treatment adherence [33]. Other variables, such as a regular supply of TB drugs, BCG vaccine, distance from health care center, alcohol use, and smoking which are frequently found in the literature to be associated with a higher risk of TB treatment default, did not reflect the increased risk for TB treatment nonadherence.

The supply of TB drugs in Kosovo is supported by Global Fund, and no interruption in drug delivery was registered until know, while the alcohol use and smoking habits were relatively small in our TB study patients.

Our results suggest that TB patient's adherence is multifactorial and cross-variate dynamic phenomenon which is influenced by several groups of medical, sociocultural, and behavioral factors [34].

To achieve more objective results on the patient's compliance with anti-TB drugs, other studies should be conducted in analyzing factors such as the TB patients' stigma, the patients and their family members, and the evaluation of patient's perception about treatment adherence.

The limitations of our study were that we did not include TB patients in the active phase of TB disease which were under direct supervision of medical staff. Moreover, the data are obtained from direct contact with TB patients and did not include the family members and medical staff who are responsible for monitoring and facilitating the TB treatment adherence. Finally, the data were analyzed by descriptive statistics and not by multivariate analysis, and we consider that as a statistical limitation.

Overall, we conclude that level of nonadherence in Kosovo patients was not satisfying and more health care worker's actions need to be addressed for improvement. The improvement of TB treatment adherence should be focused to increase the knowledge of TB disease and treatment performing more effective health education, to increase the satisfactory level of patients with health medical services, and establishing a regular monitoring system through descriptive studies to identify patients with high risk for nonadherence of TB drugs (patients living alone and other not motivated patients). Intensive health education program for TB patients and their family member should be focused to motivate the TB treatment adherence and regular visits to health facilities and to provide more information about the benefit and safety profile of TB drugs.

For additional measures we consider the organization of the modality for peer assistance TB patients working group, establishing different social communications networks to facilitate the TB treatment adherence.

Finally, we recommend more comprehensive incentives program to improve the allure of TB patients for visiting the treatment center.

## Figures and Tables

**Table 1:** Sociodemographic data http://www.sample-size.net/confidence-interval-proportion/.

Variables	Total (*n* = 324)		Adherent (*n* = 277) 95% CI (0.812–0.892)		Nonadherent (*n* = 47) 95% CI (0.109–0.188)		*p* value
	*N*	%	*n*	%	*n*	%	
Sex							
Male	160	49.38%	134	48.38%	26	55.32%	0.326
Female	164	50.62%	143	51.62%	21	44.68%	

Age (years old)							
0–15	13	4.01%	13	4.69%	0	0%	
16–25	94	29.01%	82	29.60%	12	25.53%	
26–35	40	12.35%	38	13.72%	2	4.26%	
36–45	38	11.73%	29	10.47%	9	19.15%	0.018^*∗*^
46–55	38	11.73%	30	10.83%	8	17.02%	
56–65	39	12.04%	31	11.19%	8	17.02%	
>65	62	19.14%	54	19.50%	8	17.02%	

Place of residence							
Urban	125	38.58%	100	36.10%	25	53.19%	0.015^*∗*^
Rural	199	61.42%	177	63.90%	22	46.81%	

Marital status							
Married	127	39.20%	107	38.63%	20	42.55%	0.561
Not married	194	59.88%	168	60.65%	26	55.32%	
Divorced	2	0.62%	1	0.36%	1	2.13%	
Widow	1	0.31%	1	0.36%	0	0.00%	

Education							
No education	48	14.81%	38	13.72%	10	21.28%	0.568
Elementary school	120	37.04%	104	37.55%	16	34.04%	
High school	124	38.27%	107	38.63%	17	36.17%	
University	32	9.88%	28	10.10%	4	8.51%	

Employment							
Not employed	209	64.51%	179	64.62%	30	63.83%	0.670
Employed	57	17.59%	47	16.97%	10	21.28%	
Student/pupil	58	17.90%	51	18.41%	7	14.89%	

^*∗*^ *p* < 0.05.

**Table 2:** Knowledge for TB disease.

Variables	Total (*n* = 324)		Adherent (*n* = 277) 95% CI (0.812–0.892)		Nonadherent (*n* = 47) 95% CI (0.109–0.188)		*p* value
	*N*	%	*N*	%	*n*	%	
What causes TB							
Not true	72	22.22%	64	23.10%	8	17.02%	0.407
True	70	21.60%	61	22.03%	9	19.15%	
Do not know	182	56.18%	152	54.87%	30	63.83%	

How TB is spread							
Not true	79	24.38%	67	24.19%	12	25.53%	0.207
True	121	37.35%	108	38.99%	13	27.66%	
Do not know	124	38.27%	102	36.82%	22	46.81%	

How it is prevented							
Not true	72	22.22%	62	22.38%	10	21.28%	0.864
True	90	27.78%	78	28.16%	12	25.53%	
Do not know	162	50.00%	137	49.46%	25	53.19%	

Is TB cured							
Not true	9	2.78%	5	1.81%	4	8.51%	0.003^*∗∗*^
True	286	88.27%	252	90.97%	34	72.34%	
Do not know	29	8.95%	20	7.22%	9	19.15%	

^*∗∗*^ *p* < 0.001.

**Table 3:** Knowledge for TB drugs.

Variables	Total (*n* = 324)		Adherent (*n* = 277) 95% CI (0.812–0.892)		Nonadherent (*n* = 47) 95% CI (0.109–0.188)		*p* value
	*N*	%	*n*	%	*n*	%	
Knowledge about the names of TB drugs							
Not true	22	6.79%	16	5.78%	6	12.77%	0.233
True	43	13.27%	37	13.36%	6	12.77%	
Do not know	259	79.94%	224	80.86%	35	74.46%	

Knowledge about the length of treatment of TB drugs							
Not true	19	5.86%	15	5.42%	4	8.52%	0.018^*∗*^
True	244	75.31%	216	77.98%	28	59.57%	
Do not know	61	18.83%	46	16.60%	15	31.91%	

Knowledge about the daily dosages of TB drugs							
Not true	16	4.94%	7	2.53%	9	19.15%	0.0001^*∗∗∗*^
True	90	27.78%	79	28.52%	11	23.40%	
Do not know	218	67.28%	191	68.95%	27	57.45%	

Knowledge about the colors of TB drugs							
Not true	112	34.57%	93	33.57%	19	40.44%	0.261
True	212	65.43%	184	66.43%	28	57.46%	

Knowledge about side effects of TB drugs							
Not true	249	76.85%	207	74.73%	42	89.36%	0.007^*∗∗*^
True	75	23.15%	70	25.27%	5	10.64%	

^*∗*^ *p* < 0.05; ^*∗∗*^*p* < 0.001; ^*∗∗∗*^*p* < 0.0001.

**Table 4:** Knowledge of TB drug compliance and risk behavior of TB patients.

Variables	Total (*n* = 324)		Adherent (*n* = 277) 95% CI (0.812–0.892)		Nonadherent (*n* = 47) 95% CI (0.109–0.188)		*p* value
	*N*	%	*N*	%	*n*	%	
Importance of regular administration of TB drugs							
Not true	61	18.83%	44	15.88%	17	36.17%	0.001^*∗∗*^
True	263	81.17%	233	84.12%	30	63.83%	

Administration of TB drugs in a presence of a nurse or family member							
No	202	62.35%	166	59.93%	36	76.60%	0.039^*∗*^
Yes	122	37.65%	111	40.07%	11	27.66%	

Regular supply of TB drugs							
No	87	26.85%	77	27.80%	10	21.28%	0.351
Yes	237	73.15%	200	72.20%	37	78.72%	

Satisfied with the treatment in the health center							
No	15	4.63%	6	2.17%	9	19.15%	0.0001^*∗∗∗*^
Yes	309	95.37%	271	97.83%	38	80.85%	

BCG vaccine							
No	29	8.95%	23	8.30%	6	12.77%	0.085
Yes	150	46.30%	124	44.77%	26	55.32%	
Do not know	145	44.75%	130	46.93%	15	31.91%	

Interruption of TB therapy after 3 months							
No	277	85.49%	250	90.25%	27	57.45%	0.0001^*∗∗∗*^
Yes	47	14.51%	27	9.75%	20	42.55%	

Distance from the health center							
≥10 km	102	31.48%	88	31.77%	14	29.79%	0.761
≤10 km	222	68.52%	189	68.23%	33	70.21%	

Smoking							
Yes	37	11.42%	30	10.83%	7	14.89%	0.391
No	287	88.58%	247	89.17%	40	85.11%	

Alcohol							
Yes	6	1.85%	4	1.44%	2	4.26%	0.231
No	318	98.15%	273	98.56%	45	95.74%	

^*∗*^ *p* < 0.05; ^*∗∗*^*p* < 0.001; ^*∗∗∗*^*p* < 0.0001.
